# Supplementary material for: Evidence that DNA polymerase δ contributes to initiating leading strand DNA replication in Saccharomyces cerevisiae
Source: Nat Commun. 2018 Feb 27;9:858. doi: 10.1038/s41467-018-03270-4 (PMC5829166; doi:10.1038/s41467-018-03270-4)
Supplement: Supplementary file 1 — Supplementary Information [file 41467_2018_3270_MOESM1_ESM.pdf]

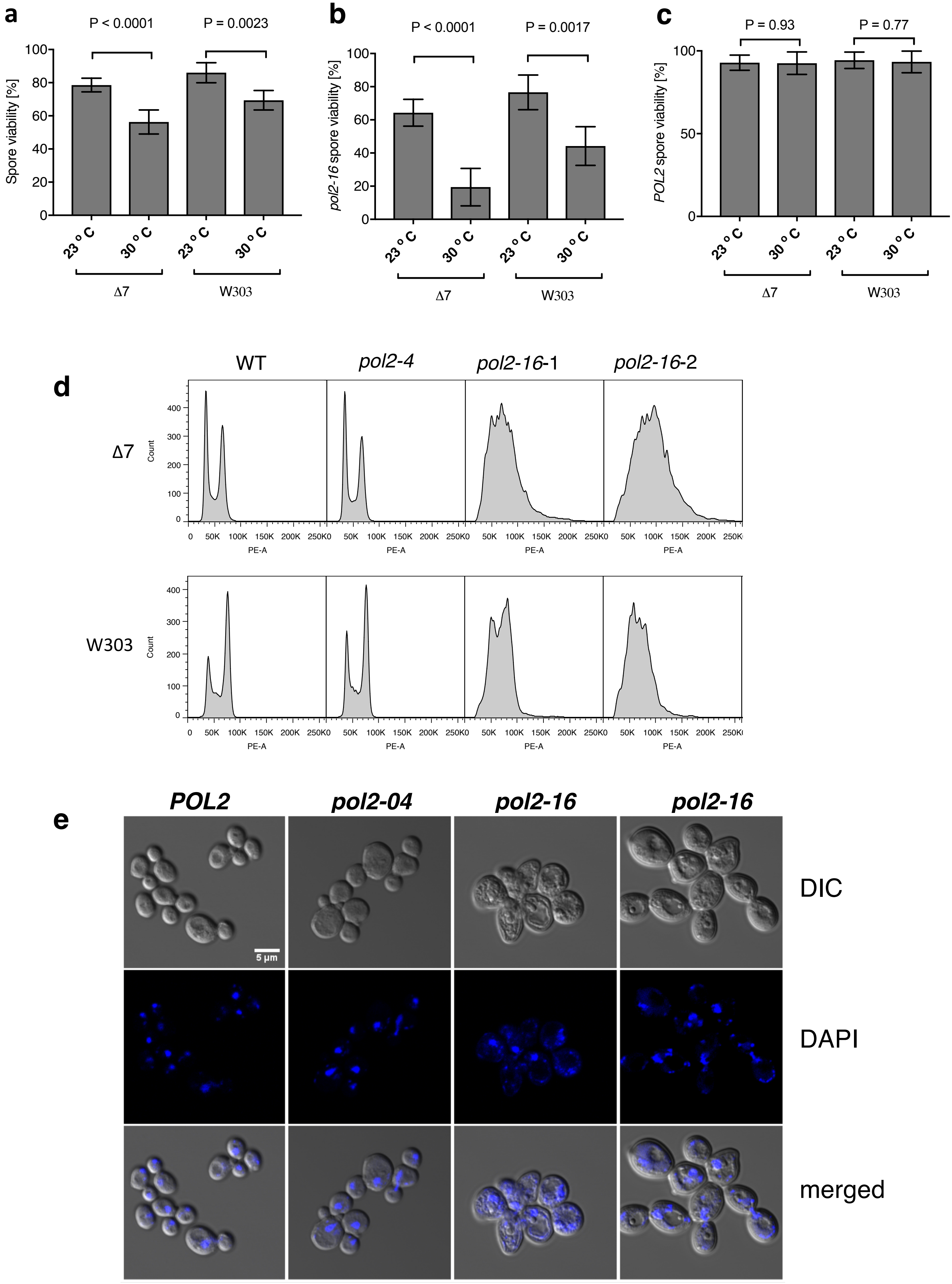
a
P < 0.0001
P = 0.0023
Spore viability [%]
0
20
40
60
80
100
23 ° C
30 ° C
23 ° C
30 ° C
Δ7
W303
b
P < 0.0001
P = 0.0017
pol2-16 spore viability [%]
0
20
40
60
80
100
23 ° C
30 ° C
23 ° C
30 ° C
Δ7
W303
c
P = 0.93
P = 0.77
POL2 spore viability [%]
0
50
100
23 ° C
30 ° C
23 ° C
30 ° C
Δ7
W303
d
WT
pol2-4
pol2-16-1
pol2-16-2
Δ7
W303
Count
0
100
200
300
400
0
50K
100K
150K
200K
250K
PE-A
e
POL2
pol2-04
pol2-16
pol2-16
5 µm
DIC
DAPI
merged

Supplementary Figure 1. Phenotypes of wild-type, *pol2-4* and *pol2-16* mutants

(a-c) Spore viability at 23° C and 30° C. For each yeast background (Δ7 and W303), 8 to 9 tetrads of 5 to 7 independent isolates were dissected and incubated at 23° C and 30° C for 12 days. The percentage of spores that were able to form colonies visible by eye were calculated. (a) total spore viability, (b) *pol2-16* spore viability and (c) wild-type spore viability. Bars represent mean +/- standard deviation (n = 5 or 7 independent yeast isolates, method: unpaired two-tailed t-test). The total spore viability and *pol2-16* spore viability were significantly lower at 30° C as compared to 23° C, because of that we decided to grow yeast at 23° C for further experiments. DNA content in exponentially growing cultures of WT, *pol2-4* and *pol2-16* strains analyzed by flow cytometry (d). Cell morphology of WT, *pol2-4* and *pol2-16* yeast strains in Δ7 background stained with DAPI (e).

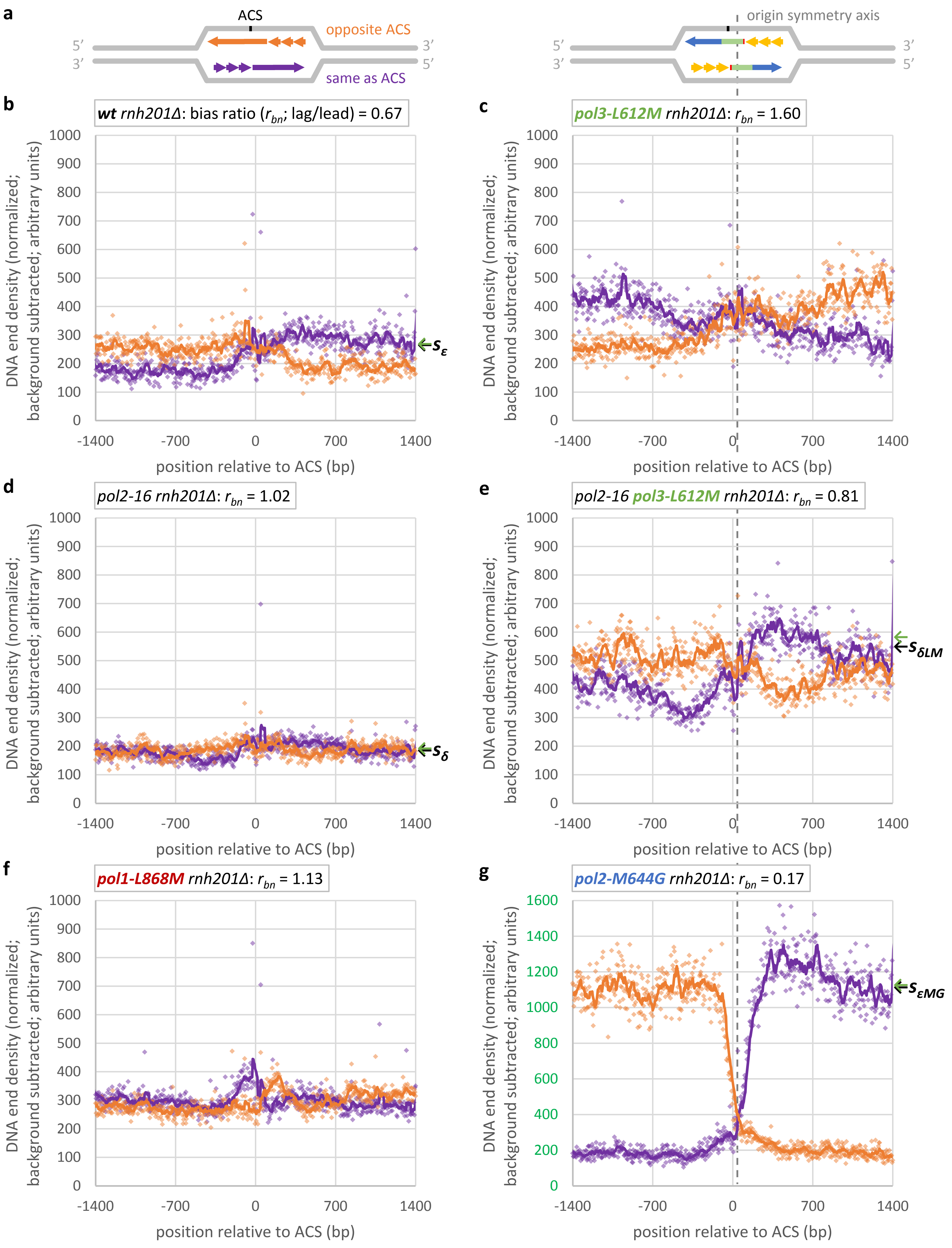

a
ACS
opposite ACS
same as ACS
origin symmetry axis
5′ 3′
3′ 5′
b
wt rnh201Δ: bias ratio (r_{bn}; lag/lead) = 0.67
s_ε
c
pol3-L612M rnh201Δ: r_{bn} = 1.60
d
pol2-16 rnh201Δ: r_{bn} = 1.02
s_δ
e
pol2-16 pol3-L612M rnh201Δ: r_{bn} = 0.81
$s_{\delta LM}$
f
pol1-L868M rnh201Δ: r_{bn} = 1.13
g
pol2-M644G rnh201Δ: r_{bn} = 0.17
$s_{\varepsilon MG}$
DNA end density (normalized; background subtracted; arbitrary units)
position relative to ACS (bp)
-1400
-700
0
700
1400

Supplementary Figure 2. **Re-normalized and background subtracted HydEn-seq end densities around *S. cerevisiae* replication origins.**

(a) Schematic representations of yeast origins. Lines represent DNA strands: parental (grey); ACS sequence (black); nascent same strand (contains the ACS; purple); nascent opposite strand (orange); continuous nascent leading strand synthesized by Pol ε (blue); canonical lagging strands synthesized by Pols α and δ (discontinuous; yellow); and non-canonical synthesis by Pols α and δ (not explained by lagging strand model; red and green, respectively). Each schematic is to scale with the following graphs. Initial regression parameters (black) are indicated by arrows next to the data from which they were estimated. The corresponding final estimates are indicated by green arrows. Green arrows indicate the corresponding final estimates. Average re-normalized lagging-over-leading strand biases (r_{bn}) are indicated above each graph (see Supplementary Methods). (b-f) Graphs of re-normalized and background subtracted HydEn-seq end densities around origins. End counts mapped to the same and opposite strands, in 5 bp bins, are colored as per panel a. Solid curves are 8-bin moving averages. Strain designations and lagging-over-leading strand bias ratios are boxed above each graph. (g) As per panels b-f, but with a different vertical scale.

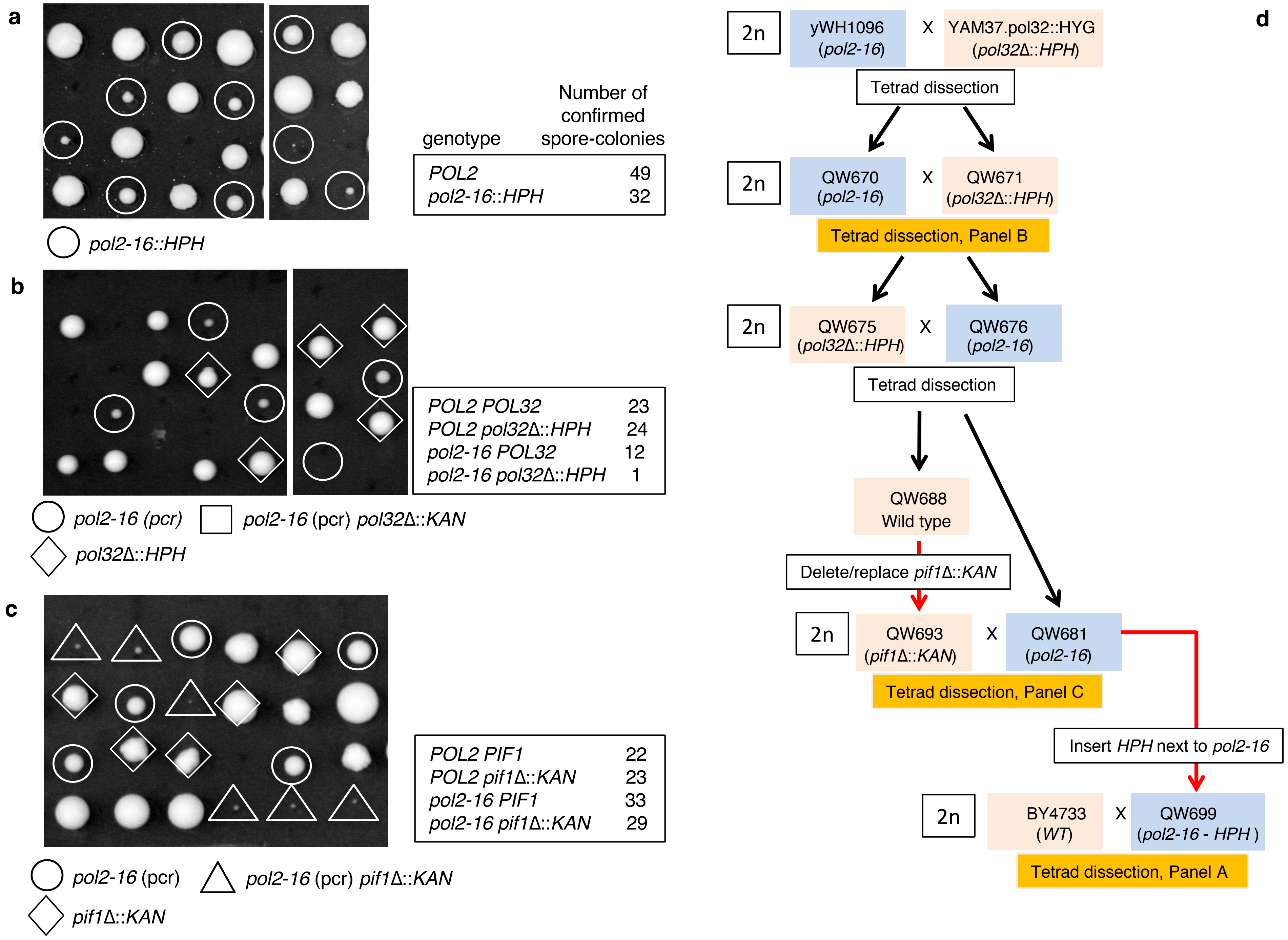

a
pol2-16::HPH
genotype
Number of confirmed spore-colonies
POL2 49
pol2-16::HPH 32
b
POL2 POL32 23
POL2 pol32Δ::HPH 24
pol2-16 POL32 12
pol2-16 pol32Δ::HPH 1
pol2-16 (pcr)
pol2-16 (pcr) pol32Δ::KAN
pol32Δ::HPH
c
POL2 PIF1 22
POL2 pif1Δ::KAN 23
pol2-16 PIF1 33
pol2-16 pif1Δ::KAN 29
pol2-16 (pcr)
pol2-16 (pcr) pif1Δ::KAN
pif1Δ::KAN
d
2n
yWH1096 (pol2-16)
X
YAM37.pol32::HYG (pol32Δ::HPH)
Tetrad dissection
2n
QW670 (pol2-16)
X
QW671 (pol32Δ::HPH)
Tetrad dissection, Panel B
2n
QW675 (pol32Δ::HPH)
X
QW676 (pol2-16)
Tetrad dissection
QW688 Wild type
Delete/replace pif1Δ::KAN
2n
QW693 (pif1Δ::KAN)
X
QW681 (pol2-16)
Tetrad dissection, Panel C
Insert HPH next to pol2-16
2n
BY4733 (WT)
X
QW699 (pol2-16 - HPH)
Tetrad dissection, Panel A

Supplementary Figure 3. Synthetic interactions of *pol32Δ* and *pif1Δ* with *pol2-16*.

The *pol2-16* mutation crossed with several strains to assess genetic interactions by tetrad analysis. (a) The segregation of *pol2-16* in an otherwise wildtype background (strain QW699 x BY4733) shows the poor growth of *pol2-16* segregants (circled). (b) When an unmarked *pol2-16* haploid (QW670) was crossed with a deletion of *POL32* (QW671), there were no viable *pol2-16 pol32Δ*::*HPH* (square) segregants, indicating synthetic lethality of this double mutant combination. Here *pol2-16* was assessed by PCR analysis of all viable segregants. (c) When *pol2-16* strain (QW681) (scored by PCR) was crossed with *pif1Δ*::*KAN* (QW693), the *pol2-16 pif1Δ*::*KAN* double mutants (diamonds) were barely viable. Number of scored spore – colonies are presented in boxes next to each tetrad dissection. (d) Strain relationships. Haploid strains propagated with *pol2-16* as the only *POL2* allele (where suppressors could be acquired), are labeled with blue boxes while all the other haploid strains are presented as light pink boxes. Tetrad dissections presented on panels a, b and c are highlighted by orange boxes. Black arrows represent lineages of segregants resulted from tetrad dissections. Red arrows were used to mark segregants modified at haploid stage.

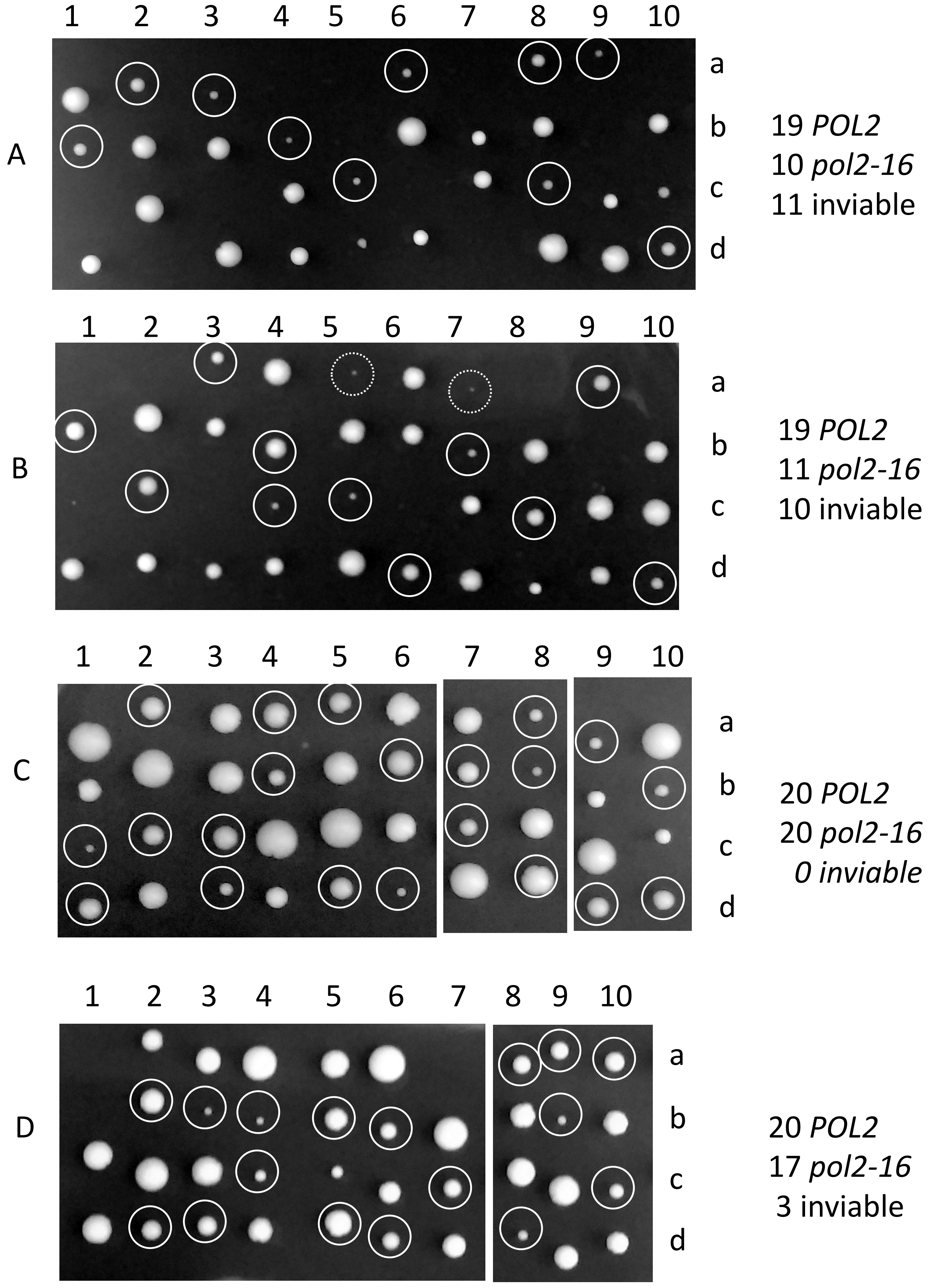
A
19 *POL2*
10 *pol2-16*
11 inviable
B
19 *POL2*
11 *pol2-16*
10 inviable
C
20 *POL2*
20 *pol2-16*
0 inviable
D
20 *POL2*
17 *pol2-16*
3 inviable
1 2 3 4 5 6 7 8 9 10
a
b
c
d

Supplementary Figure 4. Analysis of viability and colony size of the *pol2-16*/*POL2* diploid yeast (cross of BY4733 x QW710)

To support the conjecture that there is an unlinked suppressor mutation that influences the growth of *pol2-16*, we crossed QW710, a NAT-marked *pol2-16* strain, isogenic to strain QW699, and which presumably carries a suppressor of *pol2-16*, to the *POL2* strain, JY20 and obtained 14 *MAT* a *POL2* segregants that could be backcrossed to the QW710, which is *MAT* α. The resulting diploids were then sporulated and tetrads were dissected. The results of 4 such dissections are presented. In 7 of the backcrosses, the proportion of *pol2-16* segregants was approximately 50% of *POL2* segregants (examples A and B and Supplementary Table 4), whereas in at least 5 of the remaining 7 backcrosses viability of the *NAT*-marked *pol2-16* was comparable or nearly (>85%) comparable (examples C and D). The variation in the size of the colonies and the residual poor growth of some *pol2-16* segregants could still be attributed to other modifiers. *NAT*-resistant *pol2-16* segregants are circled. Two examples of very tiny colonies (size < 1 on a 0-5 scale) are shown with dotted lines.

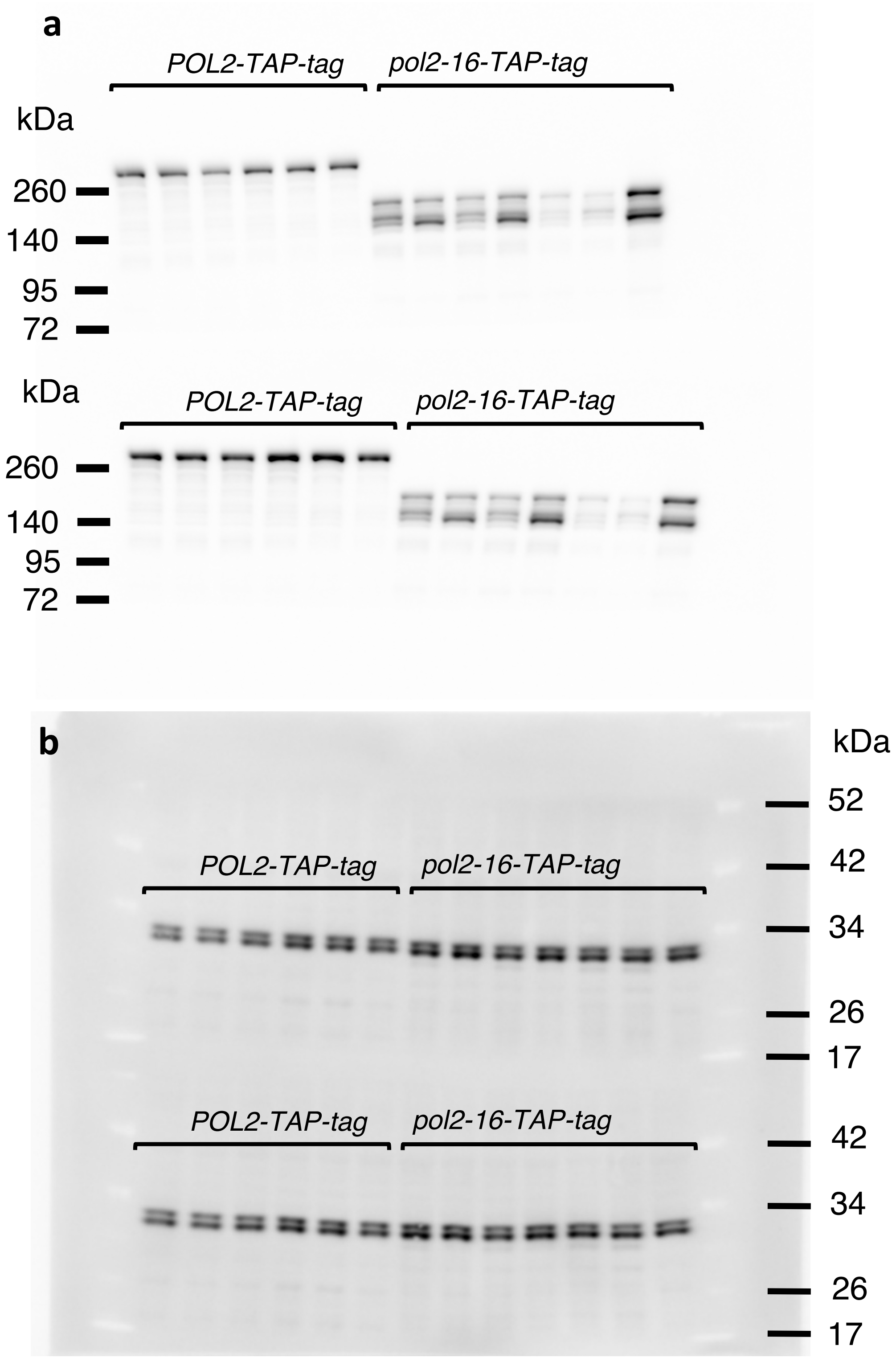

Supplementary Figure 5. Western blot detection of Pol2p level in whole cell extracts (Original images of the cropped blots shown in the Figure 1f-g)
Presented are two technical repeats for five independent isolates of strains bearing *POL2* and seven independent isolates of *pol2-16* in fusion with TAP-tag. Immunoblotting used an antibody to either TAP-tag (a) or pstair (loading control, b). Band intensities were quantified with Image Quant TL. Average intensities are presented in Figure 1g.

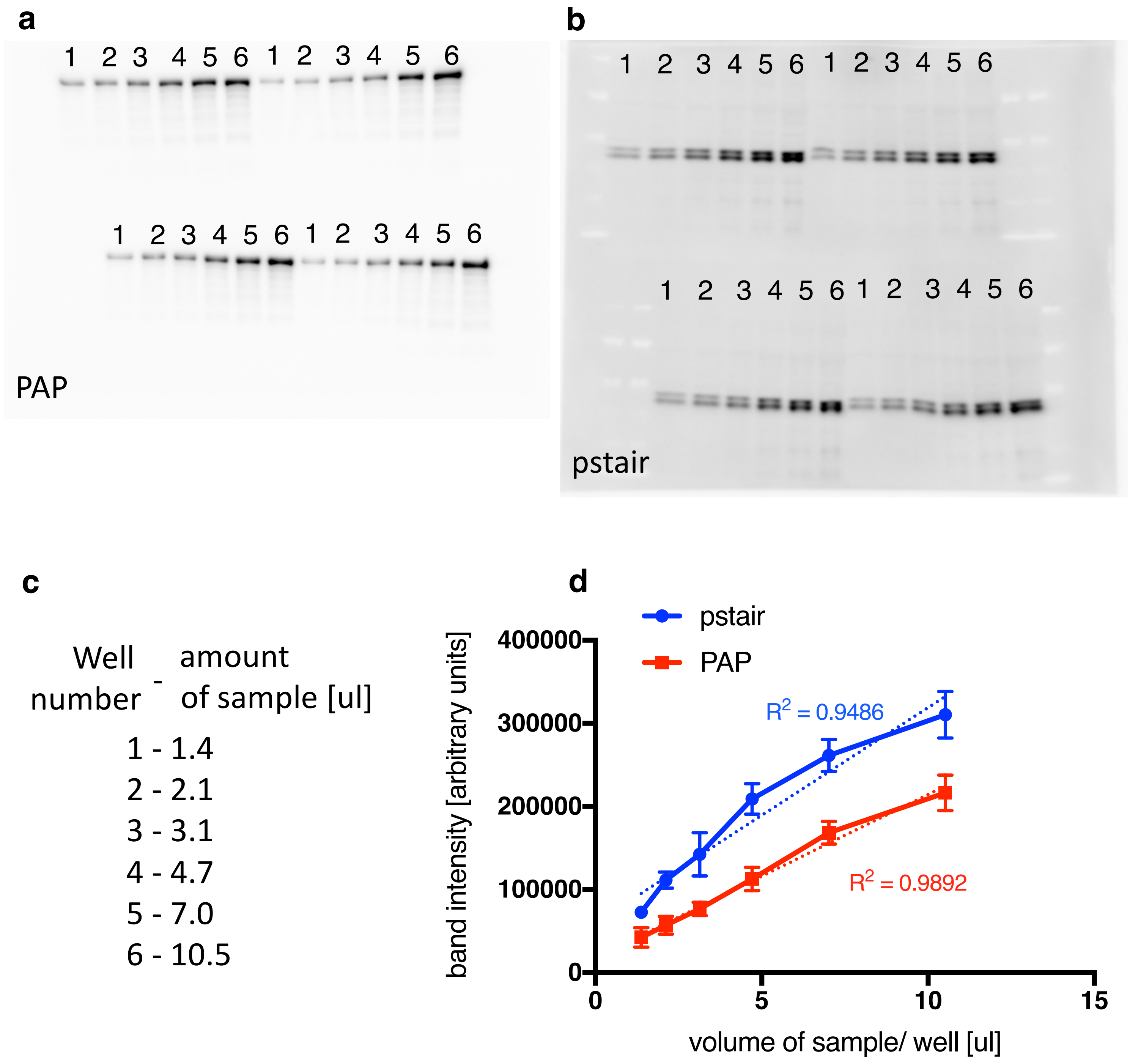

Supplementary Figure 6. Linear range of sample loading

The amount of sample loaded on the gel, both Pol2p-TAP-tag (a) and internal loading control (pstair, b), were selected from the linear range of serial dilutions (50% increase between wells) of a sample bearing Pol2p-TAP-tag (c). Bands intensities from four technical repeats (a-b) were averaged and plotted (d). Error bars represent standard deviations (n= 4).

Supplementary Table 1. Analysis of spore survival of the *pol2-16/POL2* diploid yeast in Δ7 background

Isolate Name	Temperature	alive : dead					Total of analyzed tetrads
		4 : 0	3 : 1	2 : 2	1 : 3	0 : 4	
**D1-1**	23° C	5[a]	3	0	1	0	9
	30° C	1	0	7	1	0	9
**D1-2**	23° C	3	3	2	0	0	8
	30° C	1	0	6	1	0	8
**D1-3**	23° C	2	7	0	0	0	9
	30° C	2	3	3	1	0	9
**D1-4**	23° C	3	4	0	1	0	8
	30° C	1	2	5	1	0	9
**D2-1**	23° C	2	5	2	0	0	9
	30° C	1	1	7	0	0	9
**D2-2**	23° C	4	4	1	0	0	9
	30° C	0	1	5	3	0	9
**D2-3**	23° C	2	4	3	0	0	9
	30° C	0	5	3	1	0	9
**sum of D1-(1-4)**	23° C	13[b]	17	2	2	0	34
	30° C	5	5	21	4	0	35
**sum of D2-(1-3)**	23° C	8	13	6	0	0	27
	30° C	1	7	15	4	0	27
**% of D1-(1-4)**	23° C	38%[c]	50%	6%	6%	0%	100%
	30° C	14%	14%	60%	11%	0%	100%
**% of D2-(1-3)**	23° C	30%	48%	22%	0%	0%	100%
	30° C	4%	26%	56%	15%	0%	100%

Two independent isolates of diploid wild type yeast (represented as: D1 and D2) were used to construct *pol2-16/POL2* heterozygous diploids (independent isolates are represented as: D1-1, D1-2, D1-3, D1-4 and D2-1, D2-2 and D2-3). Tetrads were dissected and incubated at 23° C for 12 days. The ratio of alive to dead spores were scored. ([a]) number of tetrads with 4 : 0 segregation; ([b]) sum of tetrads with 4 : 0 segregation in all isolates derived from parental strain D1. ([c]) fraction of tetrads with 4 : 0 segregation among all tetrads derived from parental strain D1. Based on the observed segregation we can exclude the presence of preexisting single gene suppressor in the parental yeast in Δ7 background. The presence of preexisting, homozygous,

non-linked with *POL2*, single-gene suppressor in the parental wild type diploids would manifests as 4:0 spore's segregation, which was not observed. The presence of preexisting, heterozygous, single-gene suppressor, non-linked with *POL2* in the parental wild type diploids would be possible if we observed 66% of tetrads segregate 3:1, 17% of tetrads segregate 2:2 and 17% of tetrads segregate 4:0; which was not observed in Δ7 background. In the majority of cases when the spore colony was not visible by eye there were micro-colony observed under the microscope. The spore survival ratios we observe are in better agreement with a suggestion about death of all cells at early growth stage of some *pol2-16* segregants rather than with the mono-genic suppressor(s) of *pol2-16* lethality pre-existing in the parental Δ7 background.

Supplementary Table 2. Analysis of spore survival of the *pol2-16/POL2* diploid yeast in W303 background

Isolate Name	Temperature	alive : dead					Total of analyzed tetrads
		4 : 0	3 : 1	2 : 2	1 : 3	0 : 4	
**D1-1**	23º C	5[a]	2	1	0	1	9
	30º C	2	4	3	0	0	9
**D1-2**	23º C	5	4	0	0	0	9
	30º C	1	3	5	0	0	9
**D1-3**	23º C	5	3	1	0	0	9
	30º C	3	4	2	0	0	9
**D2-1**	23º C	3	4	1	1	0	9
	30º C	ND	ND	ND	ND	ND	ND
**D2-2**	23º C	7	2	0	0	0	9
	30º C	3	2	3	1	0	9
**D2-3**	23º C	5	3	0	1	0	9
	30º C	1	3	5	0	0	9
**sum of D1-(1-3)**	23º C	15[b]	9	2	0	1	27
	30º C	6	11	10	0	0	27
**sum of D2-(1-3)**	23º C	15	9	1	2	0	27
	30º C	4	5	8	1	0	18
**% of D1-(1-3)**	23º C	56%[c]	33%	7%	0%	4%	100%
	30º C	22%	41%	37%	0%	0%	100%
**% of D2-(1-3)**	23º C	56%	33%	4%	7%	0%	100%
	30º C	22%	28%	44%	6%	0%	100%

Two independent isolates of diploid wild type yeast (represented as: D1 and D2) were used to construct *pol2-16/POL2* heterozygous diploids (independent isolates are represented as: D1-1, D1-2, D1-3 and D2-1, D2-2 and D2-3). Tetrads were dissected and incubated at 23º C for 12 days. The ratio of alive to dead spores were scored. ([a]) number of tetrads with 4 : 0 segregation; ([b]) sum of tetrads with 4 : 0 segregation in all isolates derived from parental strain D1. ([c]) fraction of tetrads with 4 : 0 segregation among all tetrads derived from parental strain D1. Based on the observed segregation we can exclude the presence of preexisting single gene suppressor in the parental yeast in W303 yeast background. The presence of preexisting, homozygous, non-linked with POL2, single-gene suppressor in the parental wild type diploids would manifests as 4:0 spore's segregation, which was not observed. The presence of preexisting, heterozygous,

single-gene suppressor, non-linked with POL2 in the parental wild type diploids would be possible if we observed 66% of tetrads segregate 3:1, 17% of tetrads segregate 2:2 and 17% of tetrads segregate 4:0; which was not observed in W303 background. In the majority of cases when the spore colony was not visible by eye there were micro-colony observed under the microscope. The spore survival ratios we observe are in better agreement with a suggestion about death of all cells at early growth stage of some *pol2-16* segregants rather than with the mono-genic suppressor(s) of *pol2-16* lethality pre-existing in the parental W303 background.

Supplementary Table 3. Analysis of spore survival of the *pol2-16/POL2* diploid yeast (cross of BY4733 x QW699)

	alive : dead					Total of analyzed tetrads
	4 : 0	3 : 1	2 : 2	1 : 3	0 : 4	
Number of tetrads	5	15	7	1	0	28
% tetrads	17.9%	53.6%	25%	3.6%	0%	100%

Tetrads of sporulated diploid yeast *pol2-16/POL2* (cross BY4733 x QW699) were dissected and incubated at 30° C for 3 days. The ratio of alive to dead spores was scored. A suppressor of *pol2-16* growth defect is apparently heterozygous in the BY4733 x QW699 diploid. If *pol2-16* and its suppressor were segregating independently there should be 4 viable : 3 viable : 2 viable in a ratio of 1 : 4: 1. The outcome is consistent with this hypothesis ($p = 0.37$; chi-square test with two degrees of freedom).

Supplementary Table 4. Analysis of viability and colony size of the *pol2-16*/*POL2* diploid yeast (cross of BY4733 x QW710)

Isolate Name	number of viable colonies*		colony size	
	POL2	*pol2-16*::NAT	*POL2*	*pol2-16*
Zygote 1	21	7 (5)	4.2	3.0
Zygote 2	21	12 (3)	4.2	1.3
Zygote 6	22	9 (2)	3.9	1.6
Zygote 12	21	10 (2)	4.3	3.3
Zygote 16	21	12 (2)	4.7	3.3
Zygote 20	21	7 (1)	4.7	3.3
Zygote 22	22	15 (2)	4.6	3.1
Zygote 3	21	17 (6)	4.1	3.2
Zygote 5	22	20 (3)	4.2	3.0
Zygote 7	21	20	4.8	3.1
Zygote 8	18	18	4.7	2.8
Zygote 10	20	16 (1)	4.6	2.7
Zygote 11	23	17 (2)	4.7	2.8
Zygote 15	17	13 (3)	4.3	2.3

Viability and colony size of backcrosses of *MAT***a** segregants from strain QW710 containing a putative growth suppressor of *pol2-16* with wild type *POL2* strain BY4733, with *MAT*α QW710. Colony size was scored on a scale of 0-5, and those with sizes ≥ 1 were counted.

* Tiny colonies, size < 1, were categorized as inviable in this scoring. See Supplementary Figure 4 for examples. The number of such colonies is given in parentheses.

Supplementary Methods

Abbreviations in calculations

Strands: lag = nascent lagging; and lead = nascent leading.

Strains: αLM = *pol1-L868M rnh201Δ*; δLM = *pol3-L612M rnh201Δ*; εMG = *pol2-M644G rnh201Δ*; ε16 = *pol2-16 rnh201Δ*; and ε16 δLM = *pol2-16 pol3-L612M rnh201Δ*.

Polymerases: α = wild type Pol α; δ = wild type Pol δ; ε = wild type Pol ε; αLM = Pol α-L868M; δ = Pol δ-L612M; and ε = Pol ε-M644G.

Lagging-over-leading strand bias calculation

The average lagging-over-leading strand bias (r_b) was calculated using ratios of RER^-/RER^+ ends mapped as same and opposite, according to the equation:

$$\text{Eq. 1:}\quad r_b = \left(\frac{\overline{x_{i,lag,K\ RER-}}}{\overline{x_{i,lag,RER+}}}\right) / \left(\frac{\overline{x_{i,lead,K\ RER-}}}{\overline{x_{i,lead,RER+}}}\right)$$

where the average end density for the leading strand in strain *K* ($\overline{x_{i,lead,K}}$) is calculated from (ACS + 750 bp) ≤ i ≤ (ACS + 1400) bp on the "same" strand as the ACS and for (ACS – 1400) bp ≤ i ≤ (ACS - 750 bp) on the "opposite" strand. The average for the lagging strand ($\overline{x_{i,lag,K}}$) is calculated from (ACS + 750 bp) ≤ i ≤ (ACS + 1400) bp on the "opposite" strand as the ACS and for (ACS – 1400) bp ≤ i ≤ (ACS - 750 bp) on the "same" strand.

Re-normalization and background subtraction

Before the HydEn-seq end densities can be used to probe the division of polymerase labor at origins, densities from different data sets must be placed on the same scale and ends that are not the intended markers for polymerase activity must be accounted for. The latter is approximated by HydEn-seq densities from strains capable of Ribonucleotide Excision Repair (RER+).

The normalized and background subtracted HydEn-seq signal for strain *K* ($y_{i,j,K}$) is thus related to the raw HydEn-seq signal ($x_{i,j,K}$) by

Eq. 2: $$y_{i,j,K} = l_K x_{i,j,K\ RER-} - b m_K x_{i,j,K\ RER+}$$

Where l_K and m_K are ratios determined empirically via comparison of end densities for strands assumed to be unaltered by relevant polymerase variants with the same strands in strains with wild type polymerases. For example, if Pol ε is the leading strand replicase distal to origins, then the leading strand end densities should be the same for *rnh201Δ*, *pol1-L868M rnh201Δ* and *pol3-L612M rnh201Δ*.

The average end density for the leading strand in strain *K* ($\overline{x_{i,lead,K}}$) is drawn from (ACS + 750 bp) ≤ *i* ≤ (ACS + 1500) bp on the same strand as the ACS and for (ACS – 1500) bp ≤ *i* ≤ (ACS - 750 bp) on the complementary ("opposite") strand. The average for the lagging strand ($\overline{x_{i,lag,K}}$) is drawn from (ACS + 750 bp) ≤ *i* ≤ (ACS + 1500) bp on the "opposite" strand as the ACS and for (ACS – 1500) bp ≤ *i* ≤ (ACS - 750 bp) on the "same" strand. Therefore

Eq. 3: $$y_{i,j,K} = \left(\frac{\overline{x_{i,lead,K\ RER-}}}{\overline{x_{i,lead,rnh201\Delta}}}\right) x_{i,j,K\ RER-} - b\left(\frac{\overline{x_{i,lead,K\ RER+}}}{\overline{x_{i,lead,WT}}}\right) x_{i,j,K\ RER+}$$

or

Eq. 4: $$y_{i,j,K} = \left(\frac{\overline{x_{i,lag,K\ RER-}}}{\overline{x_{i,lag,rnh201\Delta}}}\right) x_{i,j,K\ RER-} - b\left(\frac{\overline{x_{i,lag,K\ RER+}}}{\overline{x_{i,lag,WT}}}\right) x_{i,j,K\ RER+} \quad ,$$

depending on which polymerase gene was altered during strain construction, where *b* a factor relating RER+ and RER- strains. This factor was allowed to vary during the regression described below.

The average re-normalized lagging-over-leading strand bias (r_{bn}) is calculated as in Equation 1, but with normalized and background subtracted HydEn-seq signals:

Eq. 5: $$r_{bn} = \left(\frac{\overline{y_{i,lag,K\ RER-}}}{\overline{y_{i,lag,RER+}}}\right) / \left(\frac{\overline{y_{i,lead,K\ RER-}}}{\overline{y_{i,lead,RER+}}}\right) \quad .$$

Calculating fraction of DNA synthesized by each polymerase

Please note that regression targets (described below) explicitly assume that Pol ε is the primary leading strand replicase and that Pols α and δ synthesize the lagging strand. These assumptions preclude testing of this model of the division of polymerase labor well away from each origin, but allow acquisition of new information about the division of labor at replication origins.

To calculate the fraction of synthesis for polymerase *k* at position *i* on strand j ($f_{i,j,k}$), first assume

Eq. 6: $f_{i,j,Pol\,wild\,type} = f_{i,j,Pol\,variant}$,

Eq. 7: $1 = f_{i,j,\alpha} + f_{i,j,\varepsilon} + f_{i,j,\delta}$

and that there exists a multiplicative noise factor (*w*) that is dependent on position and strand but independent of polymerase background. Also, assume that there exists a scalar multiplier (s_k) that denotes the contribution to ribonucleotide density due to polymerase *k*. Then

Eq. 8: $y_{i,j,K} = w_{i,j} \sum_{k=1}^{n} s_k f_{i,j,k}$

and therefore

Eq. 9: $y_{i,j,\varepsilon MG} = w_{i,j}\left(s_\alpha f_{i,j,\alpha} + s_{\varepsilon MG} f_{i,j,\varepsilon} + s_\delta f_{i,j,\delta}\right)$,

Eq. 10: $y_{i,j,\delta LM} = w_{i,j}\left(s_\alpha f_{i,j,\alpha} + s_\varepsilon f_{i,j,\varepsilon} + s_{\delta LM} f_{i,j,\delta}\right)$

and

Eq. 11: $y_{i,j,\alpha LM} = w_{i,j}\left(s_{\alpha LM} f_{i,j,\alpha} + s_\varepsilon f_{i,j,\varepsilon} + s_\delta f_{i,j,\delta}\right)$.

Rearranging Eq. 9 yields

Eq. 12: $w_{i,j} = y_{i,j,\varepsilon MG} / \left(s_\alpha f_{i,j,\alpha} + s_{\varepsilon MG} f_{i,j,\varepsilon} + s_\delta f_{i,j,\delta}\right)$.

Solving Eq. 7 for f_ε yields

Eq. 13: $f_{i,j,\varepsilon} = 1 - f_{i,j,\alpha} - f_{i,j,\delta}$.

Substituting into Eqs. 7 and 8 and then solving for $f_{i,j,\alpha}$ yields

$$\text{Eq. 14: } f_{i,j,\alpha} = \frac{f_{i,j,\delta}\left(y_{i,j,\delta LM}(s_\delta - s_{\varepsilon MG}) - y_{i,j,\varepsilon MG}(s_{\delta LM} - s_\varepsilon)\right) + y_{i,j,\delta LM}s_{\varepsilon MG} - y_{i,j,\varepsilon MG}s_\varepsilon}{y_{i,j,\varepsilon MG}(s_\alpha - s_\varepsilon) - y_{i,j,\delta LM}(s_\alpha - s_{\varepsilon MG})} = \frac{f_{i,j,\delta}A + B}{C}$$

and

$$\text{Eq. 15: } f_{i,j,\alpha} = \frac{f_{i,j,\delta}\left(y_{i,j,\alpha LM}(s_\delta - s_{\varepsilon MG}) - y_{i,j,\varepsilon MG}(s_\delta - s_\varepsilon)\right) + y_{i,j,\alpha LM}s_{\varepsilon MG} - y_{i,j,\varepsilon MG}s_\varepsilon}{y_{i,j,\varepsilon MG}(s_{\alpha LM} - s_\varepsilon) - y_{i,j,\alpha LM}(s_\alpha - s_{\varepsilon MG})} = \frac{f_{i,j,\delta}D + E}{F}.$$

Setting Eq. 14 equal to Eq. 15, then solving for $f_{i,j,\delta}$ yields

$$\text{Eq. 16: } f_{i,j,\delta} = \frac{CE - FB}{FA - CD}\ .$$

Parameters were determined by least squares regression toward

$$\text{Eq. 17: } f_{i,j,\delta} = 0 \qquad ,$$

$$\text{Eq. 18: } f_{i,j,\alpha} = 0$$

and

$$\text{Eq. 19: } f_{i,j,\varepsilon} = 1$$

for (ACS + 750 bp) ≤ i ≤ (ACS + 1500) bp on the same strand as the ACS and for (ACS – 1500) bp ≤ i ≤ (ACS - 750 bp) on the complementary (“opposite”) strand. The average for these regions in strain K is $\overline{y_{i,lead,K}}$. The average for (ACS + 750 bp) ≤ i ≤ (ACS + 1500) bp on the “opposite” strand as the ACS and for (ACS – 1500) bp ≤ i ≤ (ACS - 750 bp) on the “same” strand is $\overline{y_{i,lag,K}}$.

Table 1. Polymerase participation regression parameters

Parameter	Initial Estimate	Estimate source	Constraints	Final Estimate
s_α	0.0	minimum	≥ 0	0.5
$s_{\alpha LM}$	2000.0	high guess	≥ s_α	1067.2
s_δ	184.8	$\overline{y_{i,lead,pol2-16}}$	≥ 0	190.9
$s_{\delta LM}$	559.6	$\overline{y_{i,lead,pol2-16\ \delta LM}}$	≥ s_δ	585.3

s_ε	279.0	$\overline{y_{i,lead,WT}}$	$\geq s_\delta \frac{\overline{y_{i,lead,WT}}}{\overline{y_{i,lag,WT}}}$	283.0
$s_{\varepsilon MG}$	1118.0	$\overline{y_{i,lead,\varepsilon MG}}$	$\geq s_\varepsilon$	1151.9
b	0.0	minimum	≥ 0	0.267

Constraints were selected based on observations that (1) the variant polymerases incorporate more ribonucleotides (resulting in higher HydEn-seq end densities) than due their wild type counterparts, and that (2) the leading strand bias in the *rnh201Δ* strain suggests that wild type Pol ε must have a higher ribonucleotide insertion rate than wild type Pol δ. The latter also agrees with *in vitro* data [7].

Of these, $s_{\alpha LM}$ was most sensitive to initial estimates and s_α was always unreasonably low compared to known Pol α ribonucleotide incorporation rates [7]. The contribution of Pol α during Okazaki fragment priming may be overwhelmed by, and folded into, the apparent contribution of Pol δ. This would cause underestimation of s_α and, conversely, overestimation of Pol α-derived DNA primer length.

Modeling the division of polymerase labor at replication origins

The $f_{i,j,k}$ curves suggest that replication is symmetrical about an axis of symmetry downstream of the ACS sequence and that Pol δ extends the DNA primer on the nascent leading strand until Pol ε takes over.

Least squares regression, minimizing the difference between "same" strand and the reflected "opposite" strand, suggests that the axis of symmetry is most often at position +45 bp, relative to the ACS.

An additional regression model was built to determine, probabilistically, where each polymerase begins operating on the leading strand. Three parameters defined the probability of Pol α starting leading strand DNA primer synthesis ($p_{i,j,\alpha}$): mean axis of symmetry position (μ_{sym}; fixed at + 45 bp relative to the ACS); the standard deviation in symmetry axis position

(σ_{sym}; final estimate: 58.5 bp); and the per-5-base-bin probability of Pol α starting DNA primer synthesis after departing the symmetry axis (p_{prime}; final estimate: 0.00498).

If $p_{i,s,pr}$ is the cumulative probability of priming downstream of position *i*, then

Eq. 20: $p_{i,s,pr} = \sum_{h=i}^{1500}\left(1-\left(1-p_{prime}\right)^{h-i}\right)\times n\left(h;\mu_{sym},\sigma_{sym}\right)$,

where $n(x;\mu,\sigma)$ is the normal probability density function with mean μ and standard deviation σ, *s* indicates the "same" strand as the ACS, and

Eq. 21: $p_{i,s,\alpha} = p_{i,s,pr} - p_{i-1,s,pr}$.

The probability of bin *i* being overlapped by and initial leading strand primer ($p_{i,s,\alpha ol}$) is further dependent on the fraction of the average mature Okazaki fragment synthesized by Pol α ($f_{O\alpha}$; based on an average Okazaki fragment length of 169 bp and the average of $f_{i,s,\alpha}$ for -1425 bp ≤ i ≤ -1230 bp) and the standard deviation in mature DNA primer length (σ_{pr}; final estimate: 2.69 bp). The former was used to calculate the mean primer length (μ_{pr}; final estimate: 28.8 bp):

Eq. 22: $\mu_{primer} = 169 f_{O\alpha}$.

Therefore

Eq. 23: $p_{i,s,\alpha ol} = \sum_{h=-1425}^{i}\left(p_{h-1,s,\alpha} - p_{h,s,\alpha}\right)\left(1-N\left(i-h;\mu_{pr},\sigma_{pr}\right)\right)$

Eq. 24: $p_{i,s,\delta ol} = \sum_{h=-1425}^{i} p_{h,s,\alpha}\left(1-N\left(i-h;\mu_{\delta}+\mu_{pr},\sqrt{\sigma_{pr}{}^{2}+\sigma_{\delta}{}^{2}}\right)\right) - p_{i,s,\alpha ol}$

where $N(x;\mu,\sigma)$ is the normal cumulative distribution function with mean μ and standard deviation σ.

Therefore

Eq. 25: $p_{i,s,\delta} = p_{i,s,\alpha ol} / \sum_{i=-1425}^{1500}\sum_{h=-1425}^{i}\left(p_{h-1,s,\alpha} - p_{h,s,\alpha}\right)\left(1-N\left(i-h;\mu_{pr},\sigma_{pr}\right)\right)$

and the modelled fractions of synthesis due to Pols α, δ and ε are

Eq. 26: $f_{i,s,\alpha mod} = f_{O\alpha} p_{i,s,pr} + p_{i,s,\alpha}$,

Eq. 27: $f_{i,s,\delta mod} = (1 - f_{O\alpha}) p_{i,s,pr} + p_{i,s,\delta ol}$

and

Eq. 28: $f_{i,s,\varepsilon mod} = 1 - f_{i,s,\alpha mod} - f_{i,s,\delta mod}$.
