# Supplementary material for: Evidence that DNA polymerase δ contributes to initiating leading strand DNA replication in Saccharomyces cerevisiae
Source: Nat Commun. 2018 Feb 27;9:858. doi: 10.1038/s41467-018-03270-4 (PMC5829166; doi:10.1038/s41467-018-03270-4)
Supplement: Supplementary file 3 — Description of Additional Supplementary Files [file 41467_2018_3270_MOESM3_ESM.pdf]

Description of Additional Supplementary Files

File Name: Supplementary Data 1
Description: This workbook contains five worksheets:

1. “A”: contains a list of yeast strains, their genotypes and description how they were constructed
2. “B”: contains a list of oligonucleotides used in this study
3. “C”: contains HydEnseq summary and values of SbfI-HF normalization factors
4. “D”: contains HydEnseq data sets, sum of raw read counts at 214 origins of replication per bin [5 bp]
5. “E”: contains HydEnseq data sets, sum of raw read counts at 214 origins of replication per bin [5 bp]

File Name: Supplementary Data 2
Description: This workbook contains three worksheets:

1. “rer-“: contains strand-specific HydEn-seq density measurements, summed across 214 well-characterized origins, for all Ribonucleotide Excision Repair-deficient strains (n=6) and their associated scaling factors for comparison.
2. “RER+“: contains strand-specific HydEn-seq density measurements, summed across 214 well-characterized origins, for all Ribonucleotide Excision Repair-proficient strains (n=6) and their associated scaling factors for comparison.
3. “bkgd_div_same”: contains scaled and background-subtracted HydEn-seq densities and uses these to solve a system of simultaneous equations to determine the fractional participation of each replicative polymerase at each point around the meta-origin.

File Name: Supplementary Data 3
Description: This workbook contains one worksheet:
“Sheet1“: uses the fractional participation of polymerases from “Supplementary Data 2.xlsx/bkgd_div_same” to fit a simple model of replication and thus determine such parameters as the deviation in the initial CMG binding position, the length and deviation in Pol α and Pol δ synthesis tracts, and the probability of Pol α-primase priming relative to the initial CMG position.
